# PROTOCOL: Critical appraisal of methodological quality and reporting items of systematic reviews with meta‐analysis in evidence‐based social science in China: A systematic review

**DOI:** 10.1002/cl2.1278

**Published:** 2022-09-29

**Authors:** Liping Guo, Wenjie Zhou, Xin Xing, Zhipeng Wei, Minyan Yang, Mina Ma, Kehu Yang, Howard White

**Affiliations:** ^1^ Center of Evidence‐Based Medicine, School of Basic Medicine Lanzhou University Lanzhou Gansu China; ^2^ School of Business Northwest Normal University Lanzhou Gansu China

## Abstract

This is the protocol for a Campbell systematic review. The objectives are as follows: (1) To evaluate the reporting quality of systematic reviews published in Chinese social science journals against the PRISMA and MOOSE standards; (2) To evaluate the methodology quality of systematic reviews published in Chinese social science journals against the AMSTAR‐2 and DART standards; and (3) To analyze other characteristics of systematic reviews published in Chinese social science journals using content analysis.

## 1 BACKGROUND

Evidence‐based practice has gradually developed, and more systematic reviews and meta‐analyses have been conducted in the field of social sciences in China. Systematic reviews aim to summarize “the best available research on a specific question by synthesizing the results of several studies.” They use transparent procedures to search, evaluate, and synthesize the results of relevant research whilst minimizing bias. Because of this integrative effect, systematic reviews can provide more valuable references for policy‐making and practice than individual studies. Therefore, for example, the Oxford Centre of Evidence‐Based Medicine recommended systematic review/meta‐analysis as Level A evidence (Level 1 evidence) in the evidence classification (Gates & March, 2016). The Growing What Works movement in the United Kingdom bases its evidence‐based decision‐making products on systematic reviews (Gough et al., 2018).

High‐quality systematic reviews and meta‐analyses can provide effective and scientific evidence for decision‐makers, and they are also the primary source of information for researchers to quickly grasp the current progress of a research problem (Oliver & Dickson, 2015). However, the reporting quality of a systematic review depends on its methodological rigor and the clarity of the research report. Besides, differences in methods may lead to completely opposite conclusions of systematic reviews on the same research topic (Schalken & Rietbergen, 2017; Vrieze, 2018) and even mislead subsequent researchers.

In 1987, Mulrow evaluated 50 reviews published from June 1985 to June 1986 in four major medical journals (*Annals of Internal Medicine*, *Archives of Internal Medicine*, *The Journal of the American Medical Association*, and *The New England Journal of Medicine*) and found that none of them met all eight clear scientific criteria, such as evaluating the quality of the included studies (Mulrow, 1987), Meanwhile, Sacks et al. (1987) evaluated the reporting adequacy of 83 English‐language meta‐analyses of randomized controlled trials in the medical field that published from January 1966 through October 1986, involving in six areas: study design, combinability, control of bias, statistical analysis, sensitivity analysis, and application of results. The results showed that the quality of the reports from these reviews was low, with only 1–14 items of 83 meta‐analyses being fully reported (Sacks et al., 1987). After 10 years, Sacks et al. (1996) updated the study finding that the situation had hardly improved (Sacks et al., 1996).

To improve the quality of meta‐analysis, Moher et al. (1999) issued a guideline, named Quality of Reporting of Meta‐Analysis (QUOROM), focusing on the report quality of meta‐analysis on Randomized controlled trials (Moher et al., 1999). In 2009, they revised QUOROM guidelines and renamed them Preferred Reporting Items for Systematic Reviews and Meta‐Analysis (PRISMA), which also considered the quality of reporting on systematic reviews. With the advances in systematic review methodology and terminology, Page et al. (2021) developed the PRISMA 2020 statement for reporting systematic reviews based on 60 documents with reporting guidelines for systematic reviews to generate suggested modifications to the PRISMA 2009 statement in 2021 (Page et al., 2021). Meanwhile, the number of published meta‐analyses concerning observational studies in health has increased substantially, Stroup et al. (2000) held a workshop in Atlanta Ga, in April 1997, and proposed a reporting checklist for Meta‐analysis of Observational Studies in Epidemiology (MOOSE) to examine the reporting of meta‐analyses of observational studies (Stroup et al., 2000).

In addition to the reporting checklist, Shea et al. (2009) developed a measurement tool to assess systematic reviews (AMSTAR) to evaluate the methodological quality of systematic reviews on randomized controlled trials (Shea et al., 2009). After receiving comments and feedback, the AMSTAR group revised AMSTAR and released AMSTAR‐2 in September 2017 (Shea et al., 2017), which also included non‐randomized studies of interventions (NRSI). In 2015, Diekemper et al. (2015) developed a Documentation and Appraisal Review Tool (DART) for systematic reviews, which explicitly included a quality review for biases specific to observational studies.

After these guidelines were released, more studies were conducted to explore the methodology and reporting quality of systematic reviews and meta‐analyses in medical research, such as substance abuse (Kim et al., 2021), pediatrics (Bo et al., 2020), nursing (Jin et al., 2014), orthopedics (Gagnier & Kellam, 2013), and etc.

In recent years, systematic reviews and meta‐analyses have been increasing in the field of social sciences. Social science is taken to mean any branch of academic study or science that deals with human behavior in its social and cultural aspects. It is mainly focused on the scientific study of human society and social relationships. Some researchers have already assessed the quality of systematic reviews in social science. For example, Kogut et al. (2019) examined the reporting quality of mathematics education systematic review with 40 reviews, they found deficiencies in search processes and reporting of search methods (Kogut et al., 2019). Wang et al. (2021) examined the reporting quality of 96 Campbell Systematic Reviews, finding that fewer than half (42%) were of high quality, but that quality had risen since standards were introduced (Wang et al., 2021).

In China, reviews are being undertaken in areas including Marxist Theory Studies, Management Science, Philosophy, Religion Studies, Linguistics, Law, Education, Economics, Geography, Ethnography, and Cultural Studies, Archaeology, History, Psychology, Sociology, Journalism, and Communication Studies, Political Science, Library Information Science, Sport, and Art.

Meanwhile, Chinese researchers introduced the critical appraisal methods with Chinese versions of critical appraisal tools (Ge et al., 2017; Li et al., 2009; Tao et al., 2018; Tian et al., 2015; Xiong & Chen, 2011; Zhan, 2010; Zhang et al., 2015). These tools have been widely used in systematic reviews in medical fields (Wang et al., 2015), and analyzed the methodology and reporting quality of Chinese reviews.

Tian et al. (2017) compared the methodological and reporting quality of 100 systematic reviews by authors from China and those from the United States and found them to be of similar quality (Tian et al., 2017). In 2022, Bai et al. randomly selected 200 Chinese systematic reviews in the social science field published from 2000 to 2019 in the Chinese Social Sciences Citation Index (CSSCI) database. They examined the methodological and reporting quality of these reviews and suggested that the quality of the systematic reviews was below the average level (Bai et al., 2022). However, the data source they searched was the CSSCI database which covers 500 of 2700 Chinese academic journals of social sciences, thus, it is reasonable to doubt possible selection bias in their review. Therefore, the present study aims to evaluate whether and to what extent reporting and methodology standards are met in the systematic reviews of social science in China and to assess the applicability of these tools in the Chinese context with content analysis.

## 2 OBJECTIVES

The present review includes three objectives:

1. To evaluate the reporting quality of systematic reviews published in Chinese social science journals against the PRISMA and MOOSE standards.
2. To evaluate the methodology quality of systematic reviews published in Chinese social science journals against the AMSTAR‐2 and DART standards.
3. To analyze other characteristics of systematic reviews published in Chinese social science journals using content analysis.

## 3 METHODS

### 3.1 Criteria for considering studies for this study

Completed systematic reviews and meta‐analyses published in Chinese journals between January 2009 (when PRISMA was released) and January 2022. We will include intervention reviews and observational systematic reviews with meta‐analysis in social science fields, including 19 disciplines: Marxist Theory Studies, Management Science, Philosophy, Religion Studies, Linguistics, Law, Education, Economics, Geography, Ethnography and Cultural Studies, Archaeology, History, Psychology Sociology, Journalism and Communication Studies, Political science, Library Information Science, Sport, and Art. Overviews of systematic reviews, qualitative evidence syntheses, integrative reviews, rapid reviews, and evidence syntheses/summaries are beyond the inclusion criteria. In addition, we will exclude records where only the protocol, not the final systematic review is published.

### 3.2 Search methods for identification of studies

We will search the CNKI, WanFang, and VIP databases to identify all the completed reviews published in Chinese journals from January 2009 to January 2022. The search strategy is as follows:

篇名（词）=系统评价 OR 元分析 OR 荟萃分析 OR 元综合，文献类型=论文，年=2009‐2022

(Title = systematic review OR meta‐analysis, Document types = article, Publication date = 2009‐2022).

### 3.3 Data collection and analysis *Selection of studies*

The selection of studies will be performed independently by two reviewers (Mina Ma and Minyan Yang) in Rayyan. All titles and abstracts of the records identified after retrieval will be screened, the potentially relevant references will be located with full text, and the systematic reviews that meet our criteria will be included for further analysis. Any discrepancies between the two reviewers will be resolved by consensus with another reviewer involved (Zhipeng Wei). The whole process of study screening will be reported as in the PRISMA guideline (Moher et al., 2009).

#### 3.3.1 Data extraction and management

Information extraction and coding will consist of two parts. The first is the information of publication, including the first author, title, publication year, and source of literature. The other part is the characteristics of the study content, including nine sections and 38 items (nine sections include study field, study design, title, abstract, introduction, method, result, discussion, and other information). This process will be performed by two reviewers independently (Xin Xing and Jieyun Li), and any questions will be resolved with the third author (Wenjie Zhou). Before the formal extraction, three rounds of piloting coding with 15–20 included studies will be conducted by the three authors independently using Microsoft EXCEL2019 until they reach an agreement on the extraction items. The extraction items are shown in Table 1.

**Table 1:** The items of data extraction and coding

一级类目 (The first categories)		二级类目 (The secondary category)		三级类目 (The third category)		四级类目 (The forth category)	
类目 (heading)	代码 (code)	类目 (heading)	代码 (code)	类目 (heading)	代码 (code)	类目 (heading)	代码 (code)
研究主题 (Topic)	A	马克思主义理论 (Marxist Theory Studies)	A1				
		管理学 (Management Science)	A2				
		哲学 (Philosophy)	A3				
		宗教学 (Religion Studies)	A4				
		语言学 (Linguistics)	A5				
		法学 (Law)	A6				
		教育学 (Education)	A7				
		经济学 (Economic)	A8				
		地理 (Geography)	A9				
		民族学和文化学 (Ethnology and Culturology)	A10				
		考古学 (Archaeology)	A11				
		历史 (History)	A12				
		心理学 (Psychology)	A13				
		社会学 (Sociology)	A14				
		新闻学与传播学 (Journalism and Communication Studies)	A15				
		政治学 (Political science)	A16				
		图书与情报学 (Library Information Science)	A17				
		体育学 (Sport)	A18				
		艺术学 (Art)	A19				
研究类型 (Study design)	B	观察类 (observational SR)	B1				
		干预类 (interventional SR)	B2				
题目 (Title)	C	系统评价 (systematic review)	C1				
		元分析 (meta‐analysis)	C2				
摘要 (Abstract)	D	背景 (background)	D1	理论背景 (theoretic background)	D11		
				实践背景 (practice background)	D12		
		目的 (aims)	D2	研究目的或研究问题 (purpose or research question)	D21		
		方法 (methods)	D3	纳入/排除标准 (inclusion and exclusion criteria)	D31		
				获取文献的所有来源 (literature retrieval sources)	D32		
				质量评估方法 (quality assessment method)	D33		
				结果合并方法 (data synthesis method)	D34		
		结果 (results)	D4	纳入研究数 (number of included study)	D41		
				样本量 (sample size)	D42		
				样本相关特征 (characteristics of the study)	D43		
				质量评估结果 (the results of quality assessment)	D44		
				主要结局结果 (primary outcome)	D45		
				亚组分析结果 (subgroup outcome)	D46		
		结论 (conclusions)	D5	主要研究发现 (main finding)	D51		
		讨论 (discussion)	D6	研究局限性 (limits of this review)	D61		
				结果对实践、政策和未来研究的影响 (Implications of results for practice, policy and future research)	D62		
		其他 (other information)	D7	资金来源 (funding)	D71		
				利益冲突 (conflict of interest)	D72		
				注册号 (registration number)	D73		
前言 (Introduction)	E	背景 (background)	E1	理论背景 (theoretic background)	E11		
				实践背景 (practice background)	E12		
		文献回顾 (literature review)	E2	文献回顾 (literature review)	E21		
				研究薄弱点 (limits of previous studies)	E22		
		目的 (aims)	E3	研究目的或研究问题 (purpose or research question)	E31		
		研究意义 (research meaning)	E4	理论意义 (theoretical significance)	E41		
				实践意义 (practical significance)	E42		
		研究假设 (assumptions)	E5	研究假设 (assumptions)	E51		
				研究问题 (research question)	E52		
方法 (Methods)	F	检索 (literature retrieval)	F1	获取文献的所有来源 (literature retrieval sources)	F11		
				检索截止日期 (retrieval date)	F12		
				所有数据库、注册平台和网站检索策略 (search strategy)	F13		
				手工检索 (manual search)	F14	参考文献 (reference list)	F141
						期刊 (related journals)	F142
						网页 (web page)	F143
						专家 (related experts)	F144
						其他灰色文献检索 (other grey literature search)	F145
				检索语言 (language restrictions)	F16	检索语言 (language restrictions)	F161
						排除其他语言的原因 (reason for language restrictions)	F162
				检索词 (search strategy)	F17	检索关键词 (search keywords)	F171
						检索式 (search strategy)	F172
				检索过程 (search process)	F18	检索人员数量 (number of searcher)	F181
						不一致性处理 (resolve of disagreement)	F182
				无法获取文献的处理 (treatment to unavailable literature)	F19	联系作者 (contact the author)	F191
						排除 (exclude directly)	F192
		筛选过程 (literature screen)	F2	纳入排除标准 (inclusion and exclusion criteria)	E21	研究对象 (P)	F211
						干预措施 (I)	F212
						对照措施 (C)	F213
						其他变量 (V)	F214
						结局指标 (O)	F215
						研究设计 (S)	F216
				筛选 (literature screening)	F22	筛选人员 (literature screener)	F221
						筛选过程 (screening process)	F222
						不一致性处理 (resolve of disagreement)	F223
		资料提取的方法 (data extraction and coding methods)	F3	数据提取表 (data extraction form)	F31		
				提取人员 (data extractor and coder)	F32		
				不一致性处理 (resolve of disagreement)	F33		
		数据提取资料 (Data extraction item)	F4	纳入单个研究的样本量 (sample size of each study)	F41		
				纳入研究的自变量特征 (characteristic of independent variable)	F42		
				纳入研究的因变量特征 (characteristic of dependent variable)	F43		
				纳入研究的调节变量特征 (characteristic of moderator)	F44		
				纳入研究的设计类型 (study design)	F45	随机对照试验 (RCT)	F451
						非随机对照试验 (NRCT)	
						准实验 (quasi‐experiment)	F452
						队列研究 (Cohort Studies)	F453
						病例对照研究 (case‐Control and)	F454
						横断面研究 (cross‐sectional study)	F455
				提取数据的结局指标 (outcome)	F46	平均数/标准差 (M/SD)	F461
						相关系数 (correlation coefficient)	F462
						比率 (ratio)	F463
						回归系数 (regression coefficient)	F464
						t	F466
						p	F467
						other	F468
		质量评估 (quality assessment)	F5	质量评估工具 (quality assessment tool)	F51	ROB	F511
						ROBINS	F512
						其他	F513
				质量评估人员 (rater)	F52		
				质量评估过程 (quality assessment process)	F53		
				不一致性处理 (resolve of disagreement)	F54		
		结果合并 (data synthesis methods)	F6	效应量指标 (effect size index)	F61	SMD	F611
						WMD	F612
						OR	F613
						RR	F614
						RD	F615
						Fisher's Z	F616
						Cohen's d	F617
						Hedges’ g	F618
						百分比 (percentage)	F619
				数据预处理方法 (data preparation method)	F62	缺失数据处理 (dealing with missing data)	F621
						数据转化 (data transfer)	F622
						重复数据处理 (dealing with duplicate data)	F623
				统计软件 (statistic software)	F63	RevMan	F631
						Stata	F632
						R	F633
						CMA	F634
						Excel	F635
				统计模型 (data synthesis model)	F64	随机效应模型 (random effects model)	F641
						固定效应模型 (fixed effects model)	F642
				统计模型选择理由 (reason for data synthesis model)	F65	异质性大小 (heterogeneity)	F651
						模型假设 (research purpose)	F652
				异质性检验指标 (heterogeneity index)	F66	*Q*	F661
						I^2^	F662
						Tau^2^	F663
				发表偏倚方法 (publication bias)	F67	漏斗图 (funnel plot)	F671
						Eggers检验 (Egger's test)	F672
						Begg检验 (Begg's test)	F673
						失安全系数 (fail‐safe N)	F674
						剪补法 (trim‐and‐fill)	F675
				稳健性分析 (robust analysis)	F68	敏感性分析 (sensitivity analysis)	F681
						其他 (other method)	F682
		亚组分析 (subgroup analysis)	F7	亚组分析的类型 (type of subgroup)	F71	被试特征 (characteristics of participants)	F711
						环境特征 (environment characteristic)	F712
						其他 (other)	F713
		回归分析 (regression analysis)	F8	回归分析 (regression analysis)	F81		
				结构方程模型 (structural equation model analysis)	F82		
		证据质量 (quality of evidence)	F9	证据质量分级方法 (evidence rating method)	F91		
结果 (Result)	G	研究筛选 (result of searching and screening)	G1	检索和筛选过程 (searching and screening process)	G11		
				排除的研究及被排除的原因 (reason for exclude studies)	G12		
		每个纳入研究的特征 (characteristics of included studies)	G2	描述纳入研究特征的方法 (methods of characteristics of included studies)	G21	特征表 (table)	G211
						文字描述 (text description)	G212
				纳入研究数 (number of included study)	G22		
				独立样本数 (number of included report)	G23		
				单个研究的样本量 (sample size of each study)	G24		
				自变量特征 (characteristic of independent variable)	G25		
				因变量特征 (characteristic of dependent variable)	G26		
				调节变量特征 (characteristic of moderator)	G27		
		文献质量评估结果 (the result of quality assessment)	G3	总体质量评估结果 (result of overall quality)	G31		
				单个质量评估结果 (result of individual quality)	G32		
		结果合并 (the result of data synthesis)	G4	单个研究结果呈现的方法 (method of individual result presentation)	G41	表格 (table)	G411
						森林图 (forest plot)	G412
				总的合并结果 (result of data synthesis)	G42	效应量大小 (effect size)	G421
						置信区间 (confidence interval)	G422
						异质性大小 (heterogeneity)	G423
				亚组的合并结果 (result of subgroup analysis)	G43	效应量大小 (effect size)	G431
						置信区间 (confidence interval)	G432
						异质性大小 (heterogeneity)	G433
						显著性检验 (test of significance)	G434
				发表偏倚结果 (result of publication bias)	G44	漏斗图 (funnel plot)	G441
						Egger检验 (Egger's test)	G442
						Begg检验 (Begg's test)	G443
						失安全系数 (fail‐safe N)	G444
						剪补法结果 (result of trim‐and‐fill)	G445
				稳健性分析结果 (the result of robust analysis)	G45	敏感性分析结果 (the result of sensitivity analysis)	G451
						其他 (other)	G452
		证据质量评估结果 (the result of evidence rating)		证据质量分级结果 (the result of evidence rating)	G51		
讨论 (Discussion)	H	主要发现 (main findings)	H1	主要结论 (main findings)	H11		
				与以往结论的对比 (compared with previous conclusion)	H12		
				一般性解释 (general explanation)	H13		
		调节变量分析 (findings of moderator analysis)	H2	主要结论 (main findings)	H21		
				与以往结论的对比 (compared with previous conclusion)	H22		
				一般性解释 (general explanation)	H23		
		局限性 (limitation)	H3	纳入文献局限性 (limitations of evidence)	H31		
				评价过程局限性 (limitations of review process)	H32		
				研究局限性 (limitations of study design)	H33		
		意义和启示 (significance and impact)	H4	结果对实践、政策和未来研究的影响 (Implications of results for practice, policy and future research)	H41		
其他 (Other information)	I	注册 (registration information)	I1	注册网站 (Registry platform)	I11		
				注册号 (registration number)	I12		
				对注册或计划书中所提供的信息的任何修改 (any changes to the information provided in the registration or protocol)	I13		
		财政支持和赞助 (financial support)	I2				
		利益冲突 (conflict of interest)	I3				
		作者贡献 (contribution of each author)	I4				
		数据、代码和其他材料的可获取性 (availability of data, code, and other materials)	I5				

#### 3.3.2 Quality appraisal

The reporting and methodological quality of intervention systematic reviews will be assessed using PRISMA2020 guideline and AMSTAR‐2 and observational systematic reviews will be evaluated with the MOOSE checklist DART tool. Each item of the assessment checklist will be performed in EXCEL, and the overall confidence in the results of the reviews evaluated by AMSTAR‐2 will be rated automatically on AMSTAR web. This process will be conducted independently by two authors (Xin Xing and Jieyun Li), and disagreements between coders will be resolved by discussions with another author (Wenjie Zhou). Before the formal assessment, three rounds of piloting with 15–20 included reviews will be performed to test the consistency of raters until it reaches over 95%. The items of each tool are shown in Tables 2, 3, 4, 5, 6.

**Table 2:** Items of PRISMA guideline

Section/Topic	Item	Checklist Item
**TITLE**		
Title	1	Identify the report as a systematic review.
**ABSTRACT**	2	See Table 2.
**INTRODUCTION**		
Rationale	3	Describe the rationale for the review in the context of existing knowledge.
Objectives	4	Provide an explicit statement of the objective(s) or question(s) the review addresses.
**METHODS**		
Eligibility criteria	5	Specify the inclusion and exclusion criteria for the review and how studies were grouped for the syntheses.
Information sources	6	Specify all databases, registers, websites, organizations, reference lists and other sources searched or consulted to identify studies. Specify the date when each source was last searched or consulted.
Search strategy	7	Present the full search strategies for all databases, registers and websites, including any filters and limits used.
Selection process	8	Specify the methods used to decide whether a study met the inclusion criteria of the review, including how many reviewers screened each record and each report retrieved, whether they worked independently, and if applicable, details of automation tools used in the process.
Data collection process	9	Specify the methods used to collect data from reports, including how many reviewers collected data from each report, whether they worked independently, any processes for obtaining or confirming data from study investigators, and if applicable, details of automation tools used in the process.
Data items	10a	List and define all outcomes for which data were sought. Specify whether all results that were compatible with each outcome domain in each study were sought (e.g. for all measures, time points, analyses), and if not, the methods used to decide which results to collect.
	10b	List and define all other variables for which data were sought (e.g. participant and intervention characteristics, funding sources). Describe any assumptions made about any missing or unclear information.
Study risk of bias assessment	11	Specify the methods used to assess risk of bias in the included studies, including details of the tool(s) used, how many reviewers assessed each study and whether they worked independently, and if applicable, details of automation tools used in the process.
Effect measures	12	Specify for each outcome the effect measure(s) (e.g. risk ratio, mean difference) used in the synthesis or presentation of results.
Synthesis methods	13a	Describe the processes used to decide which studies were eligible for each synthesis
	13b	Describe any methods required to prepare the data for presentation or synthesis, such as handling of missing summary statistics, or data conversions.
	13c	Describe any methods used to tabulate or visually display results of individual studies and syntheses.
	13d	Describe any methods used to synthesize results and provide a rationale for the choice(s). If meta‐analysis was performed, describe the model(s), method(s) to identify the presence and extent of statistical heterogeneity, and software package(s) used.
	13e	Describe any methods used to explore possible causes of heterogeneity among study results.
	13f	Describe any sensitivity analyses conducted to assess robustness of the synthesized results.
Reporting bias assessment	14	Describe any methods used to assess risk of bias due to missing results in a synthesis (arising from reporting biases).
Certainty assessment	15	Describe any methods used to assess certainty (or confidence) in the body of evidence for an outcome.
**RESULTS**		
Study selection	16a	Describe the results of the search and selection process, from the number of records identified in the search to the number of studies included in the review, ideally using a flow diagram.
	16b	Cite studies that met many but not all inclusion criteria (‘near‐misses’) and explain why they were excluded.
Study characteristics	17	Cite each included study and present its characteristics.
Risk of bias in studies	18	Present assessments of risk of bias for each included study.
Results of individual studies	19	For all outcomes, present, for each study: (a) summary statistics for each group (where appropriate) and (b) an effect estimates and its precision (e.g. confidence/credible interval), ideally using structured tables or plots.
Results of syntheses	20a	For each synthesis, briefly summaries the characteristics and risk of bias among contributing studies.
	20b	Present results of all statistical syntheses conducted. If meta‐analysis was done, present for each the summary estimate and its precision (e.g. confidence/credible interval) and measures of statistical heterogeneity. If comparing groups, describe the direction of the effect.
	20c	Present results of all investigations of possible causes of heterogeneity among study results.
	20d	Present results of all sensitivity analyses conducted to assess the robustness of the synthesized results.
Reporting biases	21	Present assessments of risk of bias due to missing results (arising from reporting biases) for each synthesis assessed.
Certainty of evidence	22	Present assessments of certainty (or confidence) in the body of evidence for each outcome assessed.
**DISCUSSION**		
Discussion	23a	Provide a general interpretation of the results in the context of other evidence.
	23b	Discuss any limitations of the evidence included in the review.
	23c	Discuss any limitations of the review processes used.
	23d	Discuss implications of the results for practice, policy, and future research.
**OTHER INFORMATION**		
Registration and protocol	24a	Provide registration information for the review, including register name and registration number, or state that the review was not registered.
	24b	Indicate where the review protocol can be accessed, or state that a protocol was not prepared.
	24c	Describe and explain any amendments to information provided at registration or in the protocol.
Support	25	Describe sources of financial or non‐financial support for the review, and the role of the funders or sponsors in the review.
Competing interests	26	Declare any competing interests of review authors.
Availability of data, code and other materials	27	Report which of the following are publicly available and where they can be found: template data collection forms; data extracted from included studies; data used for all analyses; analytic code; any other materials used in the review.

**Table 3:** Items of PRISMA abstract

Section/Topic	Item	Checklist item
**TITLE**		
Title	1	Identify the report as a systematic review.
**BACKGROUND**		
Objectives	2	Provide an explicit statement of the main objective(s) or question(s) the review addresses.
**METHODS**		
Eligibility criteria	3	Specify the inclusion and exclusion criteria for the review.
Information sources	4	Specify the information sources (e.g. databases, registers) used to identify studies and the date when each was last searched.
Risk of bias	5	Specify the methods used to assess risk of bias in the included studies.
Synthesis of results	6	Specify the methods used to present and synthesize results.
**RESULTS**		
Included studies	7	Give the total number of included studies and participants and summaries relevant characteristics of studies.
Synthesis of results	8	Present results for main outcomes, preferably indicating the number of included studies and participants for each. If metanalysis was done, report the summary estimate and confidence/credible interval. If comparing groups, indicate the direction of the effect (i.e. which group is favored).
**DISCUSSION**		
Limitations of evidence	9	Provide a brief summary of the limitations of the evidence included in the review (e.g. study risk of bias, inconsistency and imprecision).
Interpretation	10	Provide a general interpretation of the results and important implications.
**OTHER**		
Funding	11	Specify the primary source of funding for the review.
Registration	12	Provide the register name and registration number.

**Table 4:** Items of MOOSE checklist

Reporting Criteria	Reported (Yes/No)	Reported on Page No.
**Reporting of Background**		
1. Problem definition		
2. Hypothesis statement		
3. Description of Study Outcome(s)		
4. Type of exposure or intervention used		
5. Type of study design used		
6. Study population		
**Reporting of Search Strategy**		
7. Qualifications of searchers (e.g., librarians and investigators)		
8. Search strategy, including time period included in the synthesis and keywords		
9. Effort to include all available studies, including contact with authors		
10. Databases and registries searched		
11. Search software used, name and version, including special features used (e.g., explosion)		
12. Use of hand searching (e.g., reference lists of obtained articles)		
13. List of citations located and those excluded, including justification		
14. Method for addressing articles published in languages other than English		
15. Method of handling abstracts and unpublished studies		
16. Description of any contact with authors		
**Reporting of Methods**		
17. Description of relevance or appropriateness of studies assembled for assessing the hypothesis to be tested		
18. Rationale for the selection and coding of data (e.g., sound clinical principles or convenience)		
19. Documentation of how data were classified and coded (e.g., multiple raters, blinding, and interrater reliability)		
20. Assessment of confounding (e.g., comparability of cases and controls in studies where appropriate		
21. Assessment of study quality, including blinding of quality assessors; stratification or regression on possible predictors of study results		
22. Assessment of heterogeneity		
23. Description of statistical methods (e.g., complete description of fixed or random effects models, justification of whether the chosen models account for predictors of study results, dose‐response models, or cumulative meta‐analysis) in sufficient detail to be replicated		
24. Provision of appropriate tables and graphics		
**Reporting of results should include**		
25. Graphic summarizing individual study estimates and overall estimate		
26. Table giving descriptive information for each study included		
27. Results of sensitivity testing (e.g., subgroup analysis)		
28. Indication of statistical uncertainty of findings		
**Reporting of discussion should include**		
29. Quantitative assessment of bias (e.g., publication bias)		
30. Justification for exclusion (e.g., exclusion of non–English‐language citations)		
31. Assessment of quality of included studies		
**Reporting of conclusions should include**		
32. Consideration of alternative explanations for observed results		
33. Generalization of the conclusions (e.g., appropriate for the data presented and within the domain of the literature review)		
34. Guidelines for future research		
35. Disclosure of funding source		

**Table 5:** Items of AMSTAR‐2

Item	Checklist items	Choice			
1	Did the research questions and inclusion criteria for the review include the components of PICO?	Yes		No	
2	Did the report of the review contain an explicit statement that the review methods were established prior to the conduct of the review and did the report justify any significant deviations from the protocol?	Yes	Partial Yes	No	
3	Did the review authors explain their selection of the study designs for inclusion in the review?	Yes		No	
4	Did the review authors use a comprehensive literature search strategy?	Yes	Partial Yes	No	
5	Did the review authors perform study selection in duplicate?	Yes		No	
6	Did the review authors perform data extraction in duplicate?	Yes		No	
7	Did the review authors provide a list of excluded studies and justify the exclusions?	Yes	Partial Yes	No	
8	Did the review authors describe the included studies in adequate detail?	Yes	Partial Yes	No	
9	Did the review authors use a satisfactory technique for assessing the risk of bias (ROB) in individual studies that were included in the review?	Yes	Partial Yes	No	
10	Did the review authors report on the sources of funding for the studies included in the review?	Yes		No	
11	If meta‐analysis was performed did the review authors use appropriate methods for statistical combination of results?	Yes		No	No meta‐analysis conducted
12	If meta‐analysis was performed, did the review authors assess the potential impact of ROB in individual studies on the results of the meta‐analysis or other evidence synthesis?	Yes		No	No meta‐analysis conducted
13	Did the review authors account for ROB in individual studies when interpreting/discussing the results of the review?	Yes		No	
14	Did the review authors provide a satisfactory explanation for, and discussion of, any heterogeneity observed in the results of the review?	Yes		No	
15	If they performed quantitative synthesis did the review authors carry out an adequate investigation of publication bias (small study bias) and discuss its likely impact on the results of the review?	Yes		No	No meta‐analysis conducted
16	Did the review authors report any potential sources of conflict of interest, including any funding they received for conducting the review?	Yes		No	

**Table 6:** Items of DART tool

Item	Checklist items	Choice
1	Did the authors develop the research question(s) and inclusion/exclusion criteria before conducting the review?	
a	It was clear the authors developed the research question(s) and inclusion criteria before conducting the review and that they stated the question(s) clearly	Yes
b	Not described or cannot tell	No
2	Did the authors describe the search methods used to find evidence (original research) on the primary question(s)?	
a	Key words and/or MESH terms were stated and where feasible the search strategy was provided	Yes
b	Not described or cannot tell	No
3	Was the search for the evidence reasonably comprehensive? Were the following included?	
a	Search included at least two electronic sources	Yes/No
b	Authors chose the most applicable electronic databases (e.g., CINAHL for nursing journals, EMBASE for pharmaceutical journals, and MEDLINE for general, comprehensive search) and only limited search by date when performing an update of a previous systematic review	Yes/No
c	Search methods are likely to capture all relevant studies (e.g., includes languages other than English; gray literature such as conference proceedings, dissertations, theses, clinical trials registries and other reports) and authors hand‐searched journals or reference lists to identify published studies which were not electronically available	Yes/No
4	Did the authors do the following when selecting studies for the review?	
a	Provide in the inclusion criteria: population, intervention, outcome and study design?	Yes/No
b	State whether the selection criteria were applied independently by more than one person?	Yes/No
c	State how disagreements were resolved during study selection?	Yes/No
d	Provide a flowchart or descriptive summary of the included and excluded studies?	Yes/No
e	Include all study designs appropriate for the research questions posed?	Yes/No
5	Were the characteristics of the included studies provided? (in an aggregated form such as a table, data from the original studies were provided on the participants, interventions and outcomes)	Yes/Partially/No
6	Did the authors make any statements about assessing for publication bias?	
a	The authors did assess for publication bias and if publication bias was detected they stated how it was handled	Yes
b	The authors did assess for publication bias but did not state how it was handled if it was detected	Partially
c	Not described or cannot tell	No
7	Did the authors do the following to assess the overall quality of the individual studies included in the review?	
a	Was the quality assessment specified with adequate detail to permit replication?	Yes/No
b	Was the quality assessment conducted independently by more than one person?	Yes/No
c	Did the authors state how disagreements were resolved during the quality assessment?	Yes/No
8	Did the authors appropriately assess for quality by appropriately examining the following sources of bias in all of the included studies?	
**All studies**:		
a	Confounding (assessed comparability of study groups at start of study, was randomization successful?)	Yes/No
b	Sufficient sample size (only applicable to studies that summarize their results in a qualitative manner; it's not a concern for pooled results)	Yes/No
c	Outcome reporting bias (assessed for each outcome reported using a system such as the ORBIT classification system)	Yes/No
d	Follow up (assessed for completeness and any differential loss to follow‐up)	Yes/No
**For Randomized Controlled Trials only**:		
e	Randomization	Yes/No
f	Allocation concealment	Yes/No
g	Blinding	Yes/No
**For Case‐Control and Cohort Studies only**:		
h	Selection bias	Yes/No
i	Information bias‐‐recall and completeness to follow‐up	Yes/No
**For Quasi‐Experimental Studies only**:		
j	Differences between the first and second study measurement point ‐ such as changes or improvements in other interventions, changes in measurement techniques or definitions, or aging of subjects	Yes/No
k	Selection bias	Yes/No
**For Diagnostic Accuracy Studies only**:		
l	Selection (spectrum) bias‐were subjects selected to be representative of patients to whom the test will be applied in clinical practice, and to represent the broadest spectrum of disease?	Yes/No
m	Verification bias‐were all patients subjected to the same reference standard of diagnosis, and was it measured blindly and independently of the test?	Yes/No
9	Did the authors use appropriate methods to extract data from the included studies?	
a	Were standard forms developed and piloted prior to the systematic review conduct?	Yes/No
b	Did the authors ensure that data from the same study but that appeared in multiple publications were counted only once in the synthesis?	Yes/No
c	Was data extraction performed by more than one person?	Yes/No
10	Did the authors assess and account for heterogeneity (differences in participants, interventions, outcomes, trial design, quality or treatment effects) among the studies selected for the review?	Yes
a	The authors stated the differences among the studies and how they accounted for those differences	Partially
b	The authors stated the differences but not how they accounted for them	No
c	Not described or cannot tell	
11	Did the authors describe the methods they used to combine/synthesize the results of the relevant studies (to reach a conclusion) and were the methods used appropriate for the review question(s)?	
a	Methods were reported clearly enough to allow for replication. The overview included some assessment of the qualitative and quantitative heterogeneity of the study results and the results were appropriately combined/synthesized. For meta‐analyses, an accepted pooling method (i.e., more than simple addition) was used. Or the authors state that the evidence is conflicting and that they can't combine/synthesize the results	Yes
b	The methods were reported clearly enough to allow for replication but they were not combined appropriately	Partially
c	Not described or cannot tell	No
12	Did the authors perform sensitivity analyses on any changes in protocol, assumptions, and study selection? (For example, using sensitivity analysis to compare results from fixed effects and random effects models)	
a	Sensitivity analyses were used when appropriate on all changes in a priori design	Yes
b	Sensitivity analyses were only used on some changes in a priori design	Partially
c	Not described or cannot tell	No
13	Are the conclusions of the authors supported by the reported data with consideration of the overall quality of that data?	
a	The conclusions are supported by the reported data and reflect both the scientific quality of the studies and the risk of bias in the data obtained from those studies	Yes
b	The authors failed to consider study quality and/or their conclusions were not supported by the data, or cannot tell	No
14	Were conflicts of interest stated and were individuals excluded from the review if they reported substantial financial and intellectual conflicts of interests?	
a	Conflicts of interests were reported for each team member and individuals were excluded if they had substantial conflicts of interests	Yes
b	Conflicts of interests were reported but it was not clear whether individuals were excluded based on their conflicts of interests	Partially
c	Conflicts of interests were not reported and individuals were not excluded based on their conflicts of interests	No
15	On a scale of 1‐10, how would you judge the overall quality of the paper?	
**Rating**	Good (8‐10)	
	Fair (5‐7)	
	Poor (< 5)	

#### 3.3.3 Data synthesis

We will use the new PRISMA guideline and MOOSE checklist to reflect the reporting quality of interventional and observational systematic reviews, and descriptive statistics (frequencies and percentages) will be used to describe reporting quality of systematic reviews. We will adopt AMSTAR‐2 and DART to evaluate the methodology quality of interventional and observational systematic reviews respectively, and the results will be shown with the percentages of each grade (high, moderate, low, and critical low with AMSTAR‐2; good, fair, and poor with DART). The descriptive statistics (frequencies and percentages) will be used to describe reporting characteristics of systematic reviews based on the content analysis information.

#### 3.3.4 Planed moderators

Subgroup analyses with stratification analysis will be conducted to explore the potential difference in reporting and methodology quality depending on the source of literature and research field. The regression analysis will be performed to evaluate the differences in quality.

- **Source of literature**. According to quantitative indexes such as the influence factors of periodicals, the total number of citations, and the opinions of experts in various disciplines, the China Social Science Research and Evaluation Centre of Nanjing University developed a Chinese Social Science Citation Index (CSSCI) which included the high influence of Chinese academic journals in social science filed. We plan to compare the quality of reviews appearing in journals indexed in CSSCI and those that are not in CSSCI.
- **Research field**. We will group the reviews into 19 fields, including Marxist Theory Studies, Management Science, Philosophy, Religion Studies, Linguistics, Law, Education, Economics, Geography, Ethnography and Cultural Studies, Archaeology, History, Psychology Sociology, Journalism and Communication Studies, Political science, Library Information Science, Sport, and Art. The subgroup analysis will be used to variation in quality between different fields.
- **Publication year**. After the systematic review and meta‐analysis guidelines were released, Chinese researchers introduced the Chinese version in 2009. We will conduct a regression analysis with the publication year as the independent variable and the quality of the reviews as the dependent variable to find the change in the reporting and methodology quality of Chinese reviews in social science.

## CONTRIBUTIONS OF AUTHORS

Guo L. P. drafted the protocol, and all authors reviewed the draft and approved the final version.

## DECLARATIONS OF INTEREST

All authors declare no potential interest.

## SOURCES OF SUPPORT

**Internal sources**

• Research on the Theoretical System, International Experience, and Chinese Path of Evidence‐based Social Science, China
  Major Project of the National Social Science Fund of China

**External sources**

• There is no source supported, China

## Supporting information

Supporting information.
