# Supplementary material for: PROTOCOL: Critical appraisal of methodological quality and reporting items of systematic reviews with meta‐analysis in evidence‐based social science in China: A systematic review
Source: Campbell Syst Rev. 2022 Sep 29;18(4):e1278. doi: 10.1002/cl2.1278 (PMC9521792; doi:10.1002/cl2.1278)
Supplement: Supplementary file 1 — Supporting information. [file CL2-18-e1278-s001.docx]

Table 1. The items of data extraction and coding

| **一级类目**  **(The first categories)** | | **二级类目**  **(The secondary category)** | | **三级类目**  **(The third category)** | | **四级类目**  **(The fourth category)** | |
| --- | --- | --- | --- | --- | --- | --- | --- |
| **类目(heading)** | **代码(code)** | **类目(heading)** | **代码(code)** | **类目(heading)** | **代码(code)** | **类目(heading)** | **代码(code)** |
| 研究主题 (Topic) | A | 马克思主义理论 (Marxist Theory Studies) | A1 |  |  |  |  |
|  |  | 管理学 (Management Science) | A2 |  |  |  |  |
|  |  | 哲学 (Philosophy) | A3 |  |  |  |  |
|  |  | 宗教学(Religion Studies) | A4 |  |  |  |  |
|  |  | 语言学(Linguistics) | A5 |  |  |  |  |
|  |  | 法学 (Law) | A6 |  |  |  |  |
|  |  | 教育学 (Education) | A7 |  |  |  |  |
|  |  | 经济学 (Economic) | A8 |  |  |  |  |
|  |  | 地理 (Geography) | A9 |  |  |  |  |
|  |  | 民族学和文化学(Ethnology and Culturology) | A10 |  |  |  |  |
|  |  | 考古学(Archaeology) | A11 |  |  |  |  |
|  |  | 历史(History) | A12 |  |  |  |  |
|  |  | 心理学(Psychology) | A13 |  |  |  |  |
|  |  | 社会学(Sociology) | A14 |  |  |  |  |
|  |  | 新闻学与传播学(Journalism and Communication Studies) | A15 |  |  |  |  |
|  |  | 政治学(Political science) | A16 |  |  |  |  |
|  |  | 图书与情报学(Library Information Science) | A17 |  |  |  |  |
|  |  | 体育学(Sport) | A18 |  |  |  |  |
|  |  | 艺术学(Art) | A19 |  |  |  |  |
| 研究类型 (Study design) | B | 相关类(correlational) | B1 |  |  |  |  |
|  |  | 干预类(interventional) | B2 |  |  |  |  |
|  |  | 影响因素类(influential) | B3 |  |  |  |  |
|  |  | 现况描述类(descriptional) | B4 |  |  |  |  |
|  |  | 比率类(prevalence) | B5 |  |  |  |  |
| 题目(Title) | C | 系统评价(systematic review) | C1 |  |  |  |  |
|  |  | 元分析(meta-analysis) | C2 |  |  |  |  |
| 摘要(Abstract) | D | 背景(background) | D1 | 理论背景(theoretic background) | D11 |  |  |
|  |  |  |  | 实践背景 (practice background) | D12 |  |  |
|  |  | 目的(aims) | D2 | 研究目的或研究问题(purpose or research question) | D21 |  |  |
|  |  | 方法(methods) | D3 | 纳入/排除标准(inclusion and exclusion criteria) | D31 |  |  |
|  |  |  |  | 获取文献的所有来源 (literature retrieval sources) | D32 |  |  |
|  |  |  |  | 质量评估方法(quality assessment method) | D33 |  |  |
|  |  |  |  | 结果合并方法(data synthesis method) | D34 |  |  |
|  |  | 结果(results) | D4 | 纳入研究数 (number of included study) | D41 |  |  |
|  |  |  |  | 样本量 (sample size) | D42 |  |  |
|  |  |  |  | 样本相关特征 (characteristics of the study) | D43 |  |  |
|  |  |  |  | 质量评估结果 (the results of quality assessment ) | D44 |  |  |
|  |  |  |  | 主要结局结果 (primary outcome) | D45 |  |  |
|  |  |  |  | 亚组分析结果 (subgroup outcome) | D46 |  |  |
|  |  | 结论(conclusions) | D5 | 主要研究发现 (main finding) | D51 |  |  |
|  |  | 讨论(discussion) | D6 | 研究局限性 (limits of this review) | D61 |  |  |
|  |  |  |  | 结果对实践、政策和未来研究的影响(Implications of results for practice, policy and future research) | D62 |  |  |
|  |  | 其他(other information) | D7 | 资金来源 (funding) | D71 |  |  |
|  |  |  |  | 利益冲突 (conflict of interest) | D72 |  |  |
|  |  |  |  | 注册号 (registration number) | D73 |  |  |
| 前言(Introduction) | E | 背景(background) | E1 | 理论背景(theoretic background) | E11 |  |  |
|  |  |  |  | 实践背景 (practice background) | E12 |  |  |
|  |  | 文献回顾 (literature review) | E2 | 文献回顾(literature review) | E21 |  |  |
|  |  |  |  | 研究薄弱点(limits of previous studies) | E22 |  |  |
|  |  | 目的 (aims) | E3 | 研究目的或研究问题(purpose or research question) | E31 |  |  |
|  |  | 研究意义 (research meaning) | E4 | 理论意义(theoretical significance) | E41 |  |  |
|  |  |  |  | 实践意义(practical significance) | E42 |  |  |
|  |  | 研究假设 (assumptions) | E5 | 研究假设(assumptions) | E51 |  |  |
|  |  |  |  | 研究问题(research question) | E52 |  |  |
| 方法 (Methods) | F | 检索 (literature retrieval) | F1 | 获取文献的所有来源(literature retrieval sources) | F11 |  |  |
|  |  |  |  | 检索截止日期 (retrieval date) | F12 |  |  |
|  |  |  |  | 所有数据库、注册平台和网站检索策略 (search strategy) | F13 |  |  |
|  |  |  |  | 手工检索 (manual search) | F14 | 参考文献 (reference list) | F141 |
|  |  |  |  |  |  | 期刊 (related journals) | F142 |
|  |  |  |  |  |  | 网页 (web page) | F143 |
|  |  |  |  |  |  | 专家 (related experts) | F144 |
|  |  |  |  |  |  | 其他灰色文献检索 (other grey literature search) | F145 |
|  |  |  |  | 检索语言(language restrictions) | F16 | 检索语言(language restrictions) | F161 |
|  |  |  |  |  |  | 排除其他语言的原因 (reason for language restrictions) | F162 |
|  |  |  |  | 检索词(search strategy) | F17 | 检索关键词 (search keywords) | F171 |
|  |  |  |  |  |  | 检索式 (search strategy) | F172 |
|  |  |  |  | 检索过程 (search process) | F18 | 检索人员数量 (number of searcher) | F181 |
|  |  |  |  |  |  | 不一致性处理 (resolve of disagreement) | F182 |
|  |  |  |  | 无法获取文献的处理 (treatment to unavailable literature) | F19 | 联系作者(contact the author) | F191 |
|  |  |  |  |  |  | 排除 (exclude directly) | F192 |
|  |  | 筛选过程 (literature screen) | F2 | 纳入排除标准 (inclusion and exclusion criteria) | E21 | 研究对象(P) | F211 |
|  |  |  |  |  |  | 干预措施(I) | F212 |
|  |  |  |  |  |  | 对照措施(C) | F213 |
|  |  |  |  |  |  | 其他变量(V) | F214 |
|  |  |  |  |  |  | 结局指标(O) | F215 |
|  |  |  |  |  |  | 研究设计(S) | F216 |
|  |  |  |  | 筛选 (literature screening) | F22 | 筛选人员 (literature screener) | F221 |
|  |  |  |  |  |  | 筛选过程 (screening process) | F222 |
|  |  |  |  |  |  | 不一致性处理 (resolve of disagreement) | F223 |
|  |  | 资料提取的方法(data extraction and coding methods) | F3 | 数据提取表(data extraction form) | F31 |  |  |
|  |  |  |  | 提取人员 (data extractor and coder) | F32 |  |  |
|  |  |  |  | 不一致性处理 (resolve of disagreement) | F33 |  |  |
|  |  | 数据提取资料(Data extraction item) | F4 | 纳入单个研究的样本量 (sample size of each study) | F41 |  |  |
|  |  |  |  | 纳入研究的自变量特征 (characteristic of independent variable) | F42 |  |  |
|  |  |  |  | 纳入研究的因变量特征(characteristic of dependent variable) | F43 |  |  |
|  |  |  |  | 纳入研究的调节变量特征(characteristic of moderator) | F44 |  |  |
|  |  |  |  | 纳入研究的设计类型(study design) | F45 | 随机对照试验(RCT) | F451 |
|  |  |  |  |  |  | 非随机对照试验(NRCT) |  |
|  |  |  |  |  |  | 准实验(quasi-experiment) | F452 |
|  |  |  |  |  |  | 队列研究(Cohort Studies) | F453 |
|  |  |  |  |  |  | 病例对照研究(case-Control and) | F454 |
|  |  |  |  |  |  | 横断面研究(cross-sectional study) | F455 |
|  |  |  |  | 提取数据的结局指标(outcome) | F46 | 平均数/标准差(M/SD) | F461 |
|  |  |  |  |  |  | 相关系数(correlation coefficient ) | F462 |
|  |  |  |  |  |  | 比率 (ratio) | F463 |
|  |  |  |  |  |  | 回归系数 (regression coefficient ) | F464 |
|  |  |  |  |  |  | t | F466 |
|  |  |  |  |  |  | p | F467 |
|  |  |  |  |  |  | other | F468 |
|  |  | 质量评估(quality assessment) | F5 | 质量评估工具 (quality assessment tool) | F51 | ROB | F511 |
|  |  |  |  |  |  | ROBINS | F512 |
|  |  |  |  |  |  | 其他 | F513 |
|  |  |  |  | 质量评估人员 (rater) | F52 |  |  |
|  |  |  |  | 质量评估过程 (quality assessment process) | F53 |  |  |
|  |  |  |  | 不一致性处理 (resolve of disagreement) | F54 |  |  |
|  |  | 结果合并(data synthesis methods) | F6 | 效应量指标 (effect size index) | F61 | SMD | F611 |
|  |  |  |  |  |  | WMD | F612 |
|  |  |  |  |  |  | OR | F613 |
|  |  |  |  |  |  | RR | F614 |
|  |  |  |  |  |  | RD | F615 |
|  |  |  |  |  |  | Fisher's Z | F616 |
|  |  |  |  |  |  | Cohen’s d | F617 |
|  |  |  |  |  |  | Hedges’ g | F618 |
|  |  |  |  |  |  | 百分比(percentage) | F619 |
|  |  |  |  | 数据预处理方法 (data preparation method) | F62 | 缺失数据处理 (dealing with missing data) | F621 |
|  |  |  |  |  |  | 数据转化 (data transfer) | F622 |
|  |  |  |  |  |  | 重复数据处理 (dealing with duplicate data) | F623 |
|  |  |  |  | 统计软件 (statistic software) | F63 | RevMan | F631 |
|  |  |  |  |  |  | Stata | F632 |
|  |  |  |  |  |  | R | F633 |
|  |  |  |  |  |  | CMA | F634 |
|  |  |  |  |  |  | Excel | F635 |
|  |  |  |  | 统计模型 (data synthesis model) | F64 | 随机效应模型(random effects model) | F641 |
|  |  |  |  |  |  | 固定效应模型(fixed effects model) | F642 |
|  |  |  |  | 统计模型选择理由(reason for data synthesis model) | F65 | 异质性大小(heterogeneity) | F651 |
|  |  |  |  |  |  | 模型假设 (research purpose) | F652 |
|  |  |  |  | 异质性检验指标 (heterogeneity index) | F66 | Q | F661 |
|  |  |  |  |  |  | I^2^ | F662 |
|  |  |  |  |  |  | Tau^2^ | F663 |
|  |  |  |  | 发表偏倚方法 (publication bias) | F67 | 漏斗图 (funnel plot) | F671 |
|  |  |  |  |  |  | Eggers检验 (Egger's test) | F672 |
|  |  |  |  |  |  | Begg检验 (Begg’s test) | F673 |
|  |  |  |  |  |  | 失安全系数 (fail-safe N) | F674 |
|  |  |  |  |  |  | 剪补法 (trim-and-fill) | F675 |
|  |  |  |  | 稳健性分析 (robust analysis) | F68 | 敏感性分析 (sensitivity analysis) | F681 |
|  |  |  |  |  |  | 其他(other method) | F682 |
|  |  | 亚组分析 (subgroup analysis) | F7 | 亚组分析的类型(type of subgroup) | F71 | 被试特征 (characteristics of participants) | F711 |
|  |  |  |  |  |  | 环境特征 (environment characteristic) | F712 |
|  |  |  |  |  |  | 其他 (other) | F713 |
|  |  | 回归分析 (regression analysis) | F8 | 回归分析 (regression analysis) | F81 |  |  |
|  |  |  |  | 结构方程模型 (structural equation model analysis) | F82 |  |  |
|  |  | 证据质量(quality of evidence) | F9 | 证据质量分级方法 (evidence rating method) | F91 |  |  |
| 结果(Result) | G | 研究筛选 (result of searching and screening) | G1 | 检索和筛选过程 (searching and screening process) | G11 |  |  |
|  |  |  |  | 排除的研究及被排除的原因 (reason for exclude studies) | G12 |  |  |
|  |  | 每个纳入研究的特征 (characteristics of included studies) | G2 | 描述纳入研究特征的方法 (methods of characteristics of included studies) | G21 | 特征表(table) | G211 |
|  |  |  |  |  |  | 文字描述(text description) | G212 |
|  |  |  |  | 纳入研究数(number of included study) | G22 |  |  |
|  |  |  |  | 独立样本数 (number of included report) | G23 |  |  |
|  |  |  |  | 单个研究的样本量 (sample size of each study) | G24 |  |  |
|  |  |  |  | 自变量特征 (characteristic of independent variable) | G25 |  |  |
|  |  |  |  | 因变量特征(characteristic of dependent variable) | G26 |  |  |
|  |  |  |  | 调节变量特征(characteristic of moderator) | G27 |  |  |
|  |  | 文献质量评估结果 (the result of quality assessment) | G3 | 总体质量评估结果(result of overall quality) | G31 |  |  |
|  |  |  |  | 单个质量评估结果(result of individual quality) | G32 |  |  |
|  |  | 结果合并 (the result of data synthesis) | G4 | 单个研究结果呈现的方法 (method of individual result presentation) | G41 | 表格 (table) | G411 |
|  |  |  |  |  |  | 森林图 (forest plot) | G412 |
|  |  |  |  | 总的合并结果 (result of data synthesis) | G42 | 效应量大小 (effect size) | G421 |
|  |  |  |  |  |  | 置信区间 (confidence interval) | G422 |
|  |  |  |  |  |  | 异质性大小 (heterogeneity) | G423 |
|  |  |  |  | 亚组的合并结果 (result of subgroup analysis) | G43 | 效应量大小 (effect size) | G431 |
|  |  |  |  |  |  | 置信区间 (confidence interval) | G432 |
|  |  |  |  |  |  | 异质性大小 (heterogeneity) | G433 |
|  |  |  |  |  |  | 显著性检验 (test of significance) | G434 |
|  |  |  |  | 发表偏倚结果 (result of publication bias) | G44 | 漏斗图(funnel plot) | G441 |
|  |  |  |  |  |  | Egger检验 (Egger's test) | G442 |
|  |  |  |  |  |  | Begg检验(Begg’s test) | G443 |
|  |  |  |  |  |  | 失安全系数 (fail-safe N) | G444 |
|  |  |  |  |  |  | 剪补法结果(result of trim-and-fill) | G445 |
|  |  |  |  | 稳健性分析结果 (the result of robust analysis) | G45 | 敏感性分析结果(the result of sensitivity analysis) | G451 |
|  |  |  |  |  |  | 其他 (other) | G452 |
|  |  | 证据质量评估结果 (the result of evidence rating) |  | 证据质量分级结果 (the result of evidence rating) | G51 |  |  |
| 讨论 (Discussion) | H | 主要发现 (main findings) | H1 | 主要结论(main findings) | H11 |  |  |
|  |  |  |  | 与以往结论的对比 (compared with previous conclusion) | H12 |  |  |
|  |  |  |  | 一般性解释(general explanation) | H13 |  |  |
|  |  | 调节变量分析 (findings of moderator analysis) | H2 | 主要结论(main findings) | H21 |  |  |
|  |  |  |  | 与以往结论的对比 (compared with previous conclusion) | H22 |  |  |
|  |  |  |  | 一般性解释(general explanation) | H23 |  |  |
|  |  | 局限性 (limitation) | H3 | 纳入文献局限性(limitations of evidence) | H31 |  |  |
|  |  |  |  | 评价过程局限性(limitations of review process) | H32 |  |  |
|  |  |  |  | 研究局限性 (limitations of study design) | H33 |  |  |
|  |  | 意义和启示 (significance and impact) | H4 | 结果对实践、政策和未来研究的影响(Implications of results for practice, policy and future research) | H41 |  |  |
| 其他 (Other information) | I | 注册 (registration information) | I1 | 注册网站(Registry platform) | I11 |  |  |
|  |  |  |  | 注册号 (registration number) | I12 |  |  |
|  |  |  |  | 对注册或计划书中所提供的信息的任何修改(any changes to the information provided in the registration or protocol) | I13 |  |  |
|  |  | 财政支持和赞助 (financial support) | I2 |  |  |  |  |
|  |  | 利益冲突 (conflict of interest) | I3 |  |  |  |  |
|  |  | 作者贡献 (contribution of each author) | I4 |  |  |  |  |
|  |  | 数据、代码和其他材料的可获取性 (availability of data, code, and other materials) | I5 |  |  |  |  |

Table 2 Items of PRISMA guideline

| **Section/Topic** | **Item** | **Checklist Item** |
| --- | --- | --- |
| **TITLE** |  |  |
| Title | 1 | Identify the report as a systematic review. |
| **ABSTRACT** | 2 | See table 2. |
| **INTRODUCTION** |  |  |
| Rationale | 3 | Describe the rationale for the review in the context of existing knowledge. |
| Objectives | 4 | Provide an explicit statement of the objective(s) or question(s) the review addresses. |
| **METHODS** |  |  |
| Eligibility criteria | 5 | Specify the inclusion and exclusion criteria for the review and how studies were grouped for the syntheses. |
| Information sources | 6 | Specify all databases, registers, websites, organizations, reference lists and other sources searched or consulted to identify studies. Specify the date when each source was last searched or consulted. |
| Search strategy | 7 | Present the full search strategies for all databases, registers and websites, including any filters and limits used. |
| Selection process | 8 | Specify the methods used to decide whether a study met the inclusion criteria of the review, including how many reviewers screened each record and each report retrieved, whether they worked independently, and if applicable, details of automation tools used in the process. |
| Data collection process | 9 | Specify the methods used to collect data from reports, including how many reviewers collected data from each report, whether they worked independently, any processes for obtaining or confirming data from study investigators, and if applicable, details of automation tools used in the process. |
| Data items | 10a | List and define all outcomes for which data were sought. Specify whether all results that were compatible with each outcome domain in each study were sought (e.g. for all measures, time points, analyses), and if not, the methods used to decide which results to collect. |
|  | 10b | List and define all other variables for which data were sought (e.g. participant and intervention characteristics, funding sources). Describe any assumptions made about any missing or unclear information. |
| Study risk of bias  assessment | 11 | Specify the methods used to assess risk of bias in the included studies, including details of the tool(s) used, how many reviewers assessed each  study and whether they worked independently, and if applicable, details of automation tools used in the process. |
| Effect measures | 12 | Specify for each outcome the effect measure(s) (e.g. risk ratio, mean difference) used in the synthesis or presentation of results. |
| Synthesis methods | 13a | Describe the processes used to decide which studies were eligible for each synthesis |
|  | 13b | Describe any methods required to prepare the data for presentation or synthesis, such as handling of missing summary statistics, or data  conversions. |
|  | 13c | Describe any methods used to tabulate or visually display results of individual studies and syntheses. |
|  | 13d | Describe any methods used to synthesize results and provide a rationale for the choice(s). If meta-analysis was performed, describe the model(s), method(s) to identify the presence and extent of statistical heterogeneity, and software package(s) used. |
|  | 13e | Describe any methods used to explore possible causes of heterogeneity among study results. |
|  | 13f | Describe any sensitivity analyses conducted to assess robustness of the synthesized results. |
| Reporting bias assessment | 14 | Describe any methods used to assess risk of bias due to missing results in a synthesis (arising from reporting biases). |
| Certainty assessment | 15 | Describe any methods used to assess certainty (or confidence) in the body of evidence for an outcome. |
| **RESULTS** |  |  |
| Study selection | 16a | Describe the results of the search and selection process, from the number of records identified in the search to the number of studies included in the review, ideally using a flow diagram. |
|  | 16b | Cite studies that met many but not all inclusion criteria (‘near-misses’) and explain why they were excluded. |
| Study characteristics | 17 | Cite each included study and present its characteristics. |
| Risk of bias in studies | 18 | Present assessments of risk of bias for each included study. |
| Results of individual  studies | 19 | For all outcomes, present, for each study: (a) summary statistics for each group (where appropriate) and (b) an effect estimates and its precision (e.g. confidence/credible interval), ideally using structured tables or plots. |
| Results of syntheses | 20a | For each synthesis, briefly summaries the characteristics and risk of bias among contributing studies. |
|  | 20b | Present results of all statistical syntheses conducted. If meta-analysis was done, present for each the summary estimate and its precision (e.g. confidence/credible interval) and measures of statistical heterogeneity. If comparing groups, describe the direction of the effect. |
|  | 20c | Present results of all investigations of possible causes of heterogeneity among study results. |
|  | 20d | Present results of all sensitivity analyses conducted to assess the robustness of the synthesized results. |
| Reporting biases | 21 | Present assessments of risk of bias due to missing results (arising from reporting biases) for each synthesis assessed. |
| Certainty of evidence | 22 | Present assessments of certainty (or confidence) in the body of evidence for each outcome assessed. |
| **DISCUSSION** |  |  |
| Discussion | 23a | Provide a general interpretation of the results in the context of other evidence. |
|  | 23b | Discuss any limitations of the evidence included in the review. |
|  | 23c | Discuss any limitations of the review processes used. |
|  | 23d | Discuss implications of the results for practice, policy, and future research. |
| **OTHER INFORMATION** |  |  |
| Registration and protocol | 24a | Provide registration information for the review, including register name and registration number, or state that the review was not registered. |
|  | 24b | Indicate where the review protocol can be accessed, or state that a protocol was not prepared. |
|  | 24c | Describe and explain any amendments to information provided at registration or in the protocol. |
| Support | 25 | Describe sources of financial or non-financial support for the review, and the role of the funders or sponsors in the review. |
| Competing interests | 26 | Declare any competing interests of review authors. |
| Availability of data, code and other materials | 27 | Report which of the following are publicly available and where they can be found: template data collection forms; data extracted from included studies; data used for all analyses; analytic code; any other materials used in the review. |

Table 3 Items of PRISMA abstract

| **Section/Topic** | **Item** | **Checklist item** |
| --- | --- | --- |
| **TITLE** |  |  |
| Title | 1 | Identify the report as a systematic review. |
| **BACKGROUND** |  |  |
| Objectives | 2 | Provide an explicit statement of the main objective(s) or question(s) the review addresses. |
| **METHODS** |  |  |
| Eligibility criteria | 3 | Specify the inclusion and exclusion criteria for the review. |
| Information sources | 4 | Specify the information sources (e.g. databases, registers) used to identify studies and the date when each was last searched. |
| Risk of bias | 5 | Specify the methods used to assess risk of bias in the included studies. |
| Synthesis of results | 6 | Specify the methods used to present and synthesize results. |
| **RESULTS** |  |  |
| Included studies | 7 | Give the total number of included studies and participants and summaries relevant characteristics of studies. |
| Synthesis of results | 8 | Present results for main outcomes, preferably indicating the number of included studies and participants for each. If metanalysis was done, report the summary estimate and confidence/credible interval. If comparing groups, indicate the direction of the effect (i.e. which group is favored). |
| **DISCUSSION** |  |  |
| Limitations of  evidence | 9 | Provide a brief summary of the limitations of the evidence included in the review (e.g. study risk of bias, inconsistency and imprecision). |
| Interpretation | 10 | Provide a general interpretation of the results and important implications. |
| **OTHER** |  |  |
| Funding | 11 | Specify the primary source of funding for the review. |
| Registration | 12 | Provide the register name and registration number. |

Table 4 Items of MOOSE checklist

| **Reporting Criteria** | **Reported**  **(Yes/No)** | **Reported on Page No.** |
| --- | --- | --- |
| **Reporting of Background** |  |  |
| 1. Problem definition |  |  |
| 1. Hypothesis statement |  |  |
| 1. Description of Study Outcome(s) |  |  |
| 1. Type of exposure or intervention used |  |  |
| 1. Type of study design used |  |  |
| 1. Study population |  |  |
| **Reporting of Search Strategy** |  |  |
| 1. Qualifications of searchers (e.g., librarians and investigators) |  |  |
| 1. Search strategy, including time period included in the synthesis and keywords |  |  |
| 1. Effort to include all available studies, including contact with authors |  |  |
| 1. Databases and registries searched |  |  |
| 1. Search software used, name and version, including special features used (e.g., explosion) |  |  |
| 1. Use of hand searching (e.g., reference lists of obtained articles) |  |  |
| 1. List of citations located and those excluded, including justification |  |  |
| 1. Method for addressing articles published in languages other than English |  |  |
| 1. Method of handling abstracts and unpublished studies |  |  |
| 1. Description of any contact with authors |  |  |
| **Reporting of Methods** |  |  |
| 1. Description of relevance or appropriateness of studies assembled for assessing the hypothesis to be tested |  |  |
| 1. Rationale for the selection and coding of data (e.g., sound clinical principles or convenience) |  |  |
| 1. Documentation of how data were classified and coded (e.g., multiple raters, blinding, and interrater reliability) |  |  |
| 1. Assessment of confounding (e.g., comparability of cases and controls in studies where appropriate |  |  |
| 1. Assessment of study quality, including blinding of quality assessors; stratification or regression on possible predictors of study results |  |  |
| 1. Assessment of heterogeneity |  |  |
| 1. Description of statistical methods (e.g., complete description of fixed or random effects models, justification of whether the chosen models account for predictors of study results, dose-response models, or cumulative meta-analysis) in sufficient detail to be replicated |  |  |
| 1. Provision of appropriate tables and graphics |  |  |
| **Reporting of results should include** |  |  |
| 1. Graphic summarizing individual study estimates and overall estimate |  |  |
| 1. Table giving descriptive information for each study included |  |  |
| 1. Results of sensitivity testing (e.g., subgroup analysis) |  |  |
| 1. Indication of statistical uncertainty of findings |  |  |
| **Reporting of discussion should include** |  |  |
| 1. Quantitative assessment of bias (e.g., publication bias) |  |  |
| 1. Justification for exclusion (e.g., exclusion of non–English-language citations) |  |  |
| 1. Assessment of quality of included studies |  |  |
| **Reporting of conclusions should include** |  |  |
| 1. Consideration of alternative explanations for observed results |  |  |
| 1. Generalization of the conclusions (e.g., appropriate for the data presented and within the domain of the literature review) |  |  |
| 1. Guidelines for future research |  |  |
| 1. Disclosure of funding source |  |  |

Table 5 Items of AMSTAR-2

| **Item** | **Checklist items** | **Choice** | | | |
| --- | --- | --- | --- | --- | --- |
| 1 | Did the research questions and inclusion criteria for the review include the components of PICO? | Yes |  | No |  |
| 2 | Did the report of the review contain an explicit statement that the review methods were established prior to the conduct of the review and did the report justify any significant deviations from the protocol? | Yes | Partial Yes | No |  |
| 3 | Did the review authors explain their selection of the study designs for inclusion in the review? | Yes |  | No |  |
| 4 | Did the review authors use a comprehensive literature search strategy? | Yes | Partial Yes | No |  |
| 5 | Did the review authors perform study selection in duplicate? | Yes |  | No |  |
| 6 | Did the review authors perform data extraction in duplicate? | Yes |  | No |  |
| 7 | Did the review authors provide a list of excluded studies and justify the exclusions? | Yes | Partial Yes | No |  |
| 8 | Did the review authors describe the included studies in adequate detail? | Yes | Partial Yes | No |  |
| 9 | Did the review authors use a satisfactory technique for assessing the risk of bias (ROB) in individual studies that were included in the review? | Yes | Partial Yes | No |  |
| 10 | Did the review authors report on the sources of funding for the studies included in the review? | Yes |  | No |  |
| 11 | If meta-analysis was performed did the review authors use appropriate methods for statistical combination of results? | Yes |  | No | No meta-analysis conducted |
| 12 | If meta-analysis was performed, did the review authors assess the potential impact of ROB in individual studies on the results of the meta-analysis or other evidence synthesis? | Yes |  | No | No meta-analysis conducted |
| 13 | Did the review authors account for ROB in individual studies when interpreting/ discussing the results of the review? | Yes |  | No |  |
| 14 | Did the review authors provide a satisfactory explanation for, and discussion of, any heterogeneity observed in the results of the review? | Yes |  | No |  |
| 15 | If they performed quantitative synthesis did the review authors carry out an adequate investigation of publication bias (small study bias) and discuss its likely impact on the results of the review? | Yes |  | No | No meta-analysis conducted |
| 16 | Did the review authors report any potential sources of conflict of interest, including any funding they received for conducting the review? | Yes |  | No |  |

Table 6 Items of DART tool

| **Item** | **Checklist items** | **Choice** |
| --- | --- | --- |
| 1 | Did the authors develop the research question(s) and inclusion/exclusion criteria before conducting the review? |  |
| a | It was clear the authors developed the research question(s) and inclusion criteria before conducting the review and that they stated the question(s) clearly | Yes |
| b | Not described or cannot tell | No |
| 2 | Did the authors describe the search methods used to find evidence (original research) on the primary question(s)? |  |
| a | Key words and/or MESH terms were stated and where feasible the search strategy was provided | Yes |
| b | Not described or cannot tell | No |
| 3 | Was the search for the evidence reasonably comprehensive? Were the following included? |  |
| a | Search included at least two electronic sources | Yes/No |
| b | Authors chose the most applicable electronic databases (e.g., CINAHL for nursing journals, EMBASE for pharmaceutical journals, and MEDLINE for general, comprehensive search) and only limited search by date when performing an update of a previous systematic review | Yes/No |
| c | Search methods are likely to capture all relevant studies (e.g., includes languages other than English; gray literature such as conference proceedings, dissertations, theses, clinical trials registries and other reports) and authors hand-searched journals or reference lists to identify published studies which were not electronically available | Yes/No |
| 4 | Did the authors do the following when selecting studies for the review? |  |
| a | Provide in the inclusion criteria: population, intervention, outcome and study design? | Yes/No |
| b | State whether the selection criteria were applied independently by more than one person? | Yes/No |
| c | State how disagreements were resolved during study selection? | Yes/No |
| d | Provide a flowchart or descriptive summary of the included and excluded studies? | Yes/No |
| e | Include all study designs appropriate for the research questions posed? | Yes/No |
| 5 | Were the characteristics of the included studies provided? (in an aggregated form such as a table, data from the original studies were provided on the participants, interventions and outcomes) | Yes/ Partially/ No |
| 6 | Did the authors make any statements about assessing for publication bias? |  |
| a | The authors did assess for publication bias and if publication bias was detected they stated how it was handled | Yes |
| c | The authors did assess for publication bias but did not state how it was handled if it was detected | Partially |
| d | Not described or cannot tell | No |
| 7 | Did the authors do the following to assess the overall quality of the individual studies included in the review? |  |
| a | Was the quality assessment specified with adequate detail to permit replication? | Yes/No |
| c | Was the quality assessment conducted independently by more than one person? | Yes/No |
| c | Did the authors state how disagreements were resolved during the quality assessment? | Yes/No |
| 8 | Did the authors appropriately assess for quality by appropriately examining the following sources of bias in all of the included studies? |  |
| **All studies:** | |  |
| a | Confounding (assessed comparability of study groups at start of study, was randomization successful?) | Yes/No |
| b | Sufficient sample size (only applicable to studies that summarize their results in a qualitative manner; it's not a concern for pooled results) | Yes/No |
| c | Outcome reporting bias (assessed for each outcome reported using a system such as the ORBIT classification system) | Yes/No |
| d | Follow up (assessed for completeness and any differential loss to follow-up) | Yes/No |
| **For Randomized Controlled Trials only:** | |  |
| e | Randomization | Yes/No |
| f | Allocation concealment | Yes/No |
| g | Blinding | Yes/No |
| **For Case-Control and Cohort Studies only:** | |  |
| h | Selection bias | Yes/No |
| i | Information bias--recall and completeness to follow-up | Yes/No |
| **For Quasi-Experimental Studies only:** | |  |
| j | Differences between the first and second study measurement point - such as changes or improvements in other interventions, changes in measurement techniques or definitions, or aging of subjects | Yes/No |
| k | Selection bias | Yes/No |
| **For Diagnostic Accuracy Studies only:** | |  |
| l | Selection (spectrum) bias-were subjects selected to be representative of patients to whom the test will be applied in clinical practice, and to represent the broadest spectrum of disease? | Yes/No |
| m | Verification bias-were all patients subjected to the same reference standard of diagnosis, and was it measured blindly and independently of the test? | Yes/No |
| 9 | Did the authors use appropriate methods to extract data from the included studies? |  |
| a | Were standard forms developed and piloted prior to the systematic review conduct? | Yes/No |
| b | Did the authors ensure that data from the same study but that appeared in multiple publications were counted only once in the synthesis? | Yes/No |
| c | Was data extraction performed by more than one person? | Yes/No |
| 10 | Did the authors assess and account for heterogeneity (differences in participants, interventions, outcomes, trial design, quality or treatment effects) among the studies selected for the review? | Yes |
| a | The authors stated the differences among the studies and how they accounted for those differences | Partially |
| b | The authors stated the differences but not how they accounted for them | No |
| c | Not described or cannot tell |  |
| 11 | Did the authors describe the methods they used to combine/synthesize the results of the relevant studies (to reach a conclusion) and were the methods used appropriate for the review question(s)? |  |
| a | Methods were reported clearly enough to allow for replication. The overview included some assessment of the qualitative and quantitative heterogeneity of the study results and the results were appropriately combined/synthesized. For meta-analyses, an accepted pooling method (i.e., more than simple addition) was used. Or the authors state that the evidence is conflicting and that they can't combine/synthesize the results | Yes |
| b | The methods were reported clearly enough to allow for replication but they were not combined appropriately | Partially |
| c | Not described or cannot tell | No |
| 12 | Did the authors perform sensitivity analyses on any changes in protocol, assumptions, and study selection? (For example, using sensitivity analysis to compare results from fixed effects and random effects models) |  |
| a | Sensitivity analyses were used when appropriate on all changes in a priori design | Yes |
| b | Sensitivity analyses were only used on some changes in a priori design | Partially |
| c | Not described or cannot tell | No |
| 13 | Are the conclusions of the authors supported by the reported data with consideration of the overall quality of that data? |  |
| a | The conclusions are supported by the reported data and reflect both the scientific quality of the studies and the risk of bias in the data obtained from those studies | Yes |
| b | The authors failed to consider study quality and/or their conclusions were not supported by the data, or cannot tell | No |
| 14 | Were conflicts of interest stated and were individuals excluded from the review if they reported substantial financial and intellectual conflicts of interests? |  |
| a | Conflicts of interests were reported for each team member and individuals were excluded if they had substantial conflicts of interests | Yes |
| b | Conflicts of interests were reported but it was not clear whether individuals were excluded based on their conflicts of interests | Partially |
| c | Conflicts of interests were not reported and individuals were not excluded based on their conflicts of interests | No |
| 15 | On a scale of 1-10, how would you judge the overall quality of the paper? |  |
| Rating | Good (8-10) |  |
|  | Fair (5-7) |  |
|  | Poor (< 5) |  |
